# Thyroid cancer causing obstruction of the great veins in the neck

**DOI:** 10.1186/1477-7819-6-40

**Published:** 2008-04-18

**Authors:** Steve L Hyer, Prasad Dandekar, Kate Newbold, Masud Haq, Kshama Wechalakar, Khin Thway, Clive Harmer

**Affiliations:** 1 Thyroid Unit, Royal Marsden Hospital, Fulham Road, London, SW36JJ, UK; 2 Department of Pathology, Royal Marsden Hospital, Fulham Road, London, SW3 6JJ, UK; 3 St Helier Hospital, Wrythe Lane, Carshalton, Surrey, SM5 1AA, UK

## 

After publication of this work [1], we noted that we inadvertently failed to include the complete list of all coauthors. The full list of authors has now been added and the Authors' contributions and Competing interests section modified accordingly.

## Competing interests

The authors declare that they have no competing interests.

## Authors' contributions

SLH: Final draft and literature review; PD: Clinical information, initial draft, KN: Discussion and editing, MH: Clinical information, CT images and interpretation, KT: Pathological images and reports, KW: Scintigram images and interpretation, CH: Original concept, final editing

All authors read and approved final manuscript
